# *Tropheryma whipplei *tricuspid endocarditis: a case report and review of the literature

**DOI:** 10.1186/1752-1947-4-245

**Published:** 2010-08-04

**Authors:** Vincent Gabus, Zita Grenak-Degoumois, Severin Jeanneret, Riana Rakotoarimanana, Gilbert Greub, Daniel Genné

**Affiliations:** 1 Department of Internal Medicine, Centre Hospitalier Universitaire Vaudois and University of Lausanne, Switzerland; 2 Department of Internal Medicine, Community Hospital, 2300 La Chaux-de-Fonds, Switzerland; 3 Infectious disease service, Centre Hospitalier Universitaire Vaudois and University of Lausanne, Switzerland; 4 Institute of Microbiology, Centre Hospitalier Universitaire Vaudois and University of Lausanne, Switzerland

## Abstract

**Introduction:**

The main clinical manifestations of Whipple's disease are weight loss, arthropathy, diarrhea and abdominal pain. Cardiac involvement is frequently described. However, endocarditis is rare and is not usually the initial presentation of the disease. To the best of our knowledge, this is the first reported case of a patient with *Tropheryma whipplei *tricuspid endocarditis without any other valve involved and not presenting signs of arthralgia and abdominal involvement.

**Case presentation:**

We report a case of a 50-year-old Caucasian man with tricuspid endocarditis caused by *Tropheryma whipplei*, showing signs of severe shock and an absence of other more classic clinical signs of Whipple's disease, such as arthralgia, abdominal pain and diarrhea. *Tropheryma whipplei *was documented by polymerase chain reaction of the blood and pleural fluid. The infection was treated with a combined treatment of doxycycline, hydroxychloroquine and sulfamethoxazole-trimethoprim for one year.

**Conclusion:**

*Tropheryma whipplei *infectious endocarditis should always be considered when facing a blood-culture negative endocarditis particularly in right-sided valves. Although not standardized yet, treatment of *Tropheryma whipplei *endocarditis should probably include a bactericidal antibiotic (such as doxycycline) and should be given over a prolonged period of time (a minimum of one year).

## Introduction

The Gram positive bacillus *Tropheryma whippelii *was first characterized by polymerase chain reaction (PCR) in the early 1990s [1], and renamed *Tropheryma whipplei *in 2001 after its first culture and characterization [2]. The main clinical manifestations of Whipple's disease are weight loss (in 80 to 90% of reported cases), arthropathy (70 to 90%), diarrhea (70 to 85%) and abdominal pain (50 to 90%) [3]. Cardiac involvement is reported in 17 to 55% of patients with classical Whipple's disease, pericarditis being the most frequent [4]. Endocarditis, however, is rare and 88% of cases occur in patients with healthy valves without underlying disease [5]. Endocarditis was the initial presentation of only a few cases [6-10]. We report a case of a patient with tricuspid endocarditis due to *Tropheryma whipplei *and review all previously reported cases.

## Case presentation

**Figure 1:**
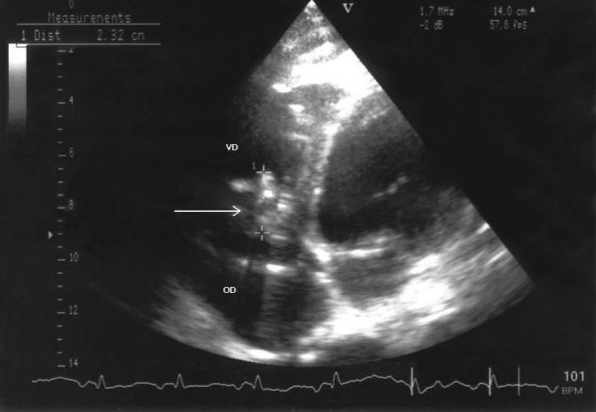
**Transthoracic echocardiography at time of diagnosis showing large vegetations on the tricuspid valve caused by *Tropheryma whipplei***. The tricuspid valve is indicated by an arrow. VD; right ventricle: OD; right atrium.

**Figure 2:**
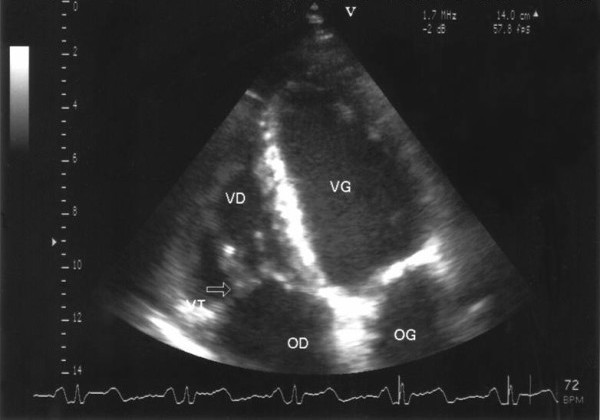
**Transthoracic echocardiography after one year of treatment**. No vegetation was found. The tricuspid valve is indicated by an arrow. VD; right ventricle: OD; right atrium: VG; left ventricle: OG; left atrium.

A 50-year-old Caucasian alcoholic man presented to the emergency department with generalized weakness lasting 10 days and a history of weight loss. He had no other complaints. His history was significant for excessive alcohol intake and cachexia. At the emergency department, the patient was weak but alert, appeared ill and was very pale. The clinical exam revealed: a temperature of 35.9°C, blood pressure 60/38 mm Hg; a heart rate of 95 beats per minute, a respiratory rate of 23 breaths per minute, bilateral ankle edema, buccal candidiasis, and a faint systolic murmur. The neurological exam was normal, except for psychomotor slowing and a fine tremor. Laboratory results showed: hemoglobin 42 g/l, platelet count 23 G/l, WBC 4.9 G/l (normally distributed), C-reactive protein 21 mg/l (N < 5), hypoalbuminaemia and cholestasis. Other laboratory tests were normal. A chest radiograph showed cardiomegaly and pulmonary vascular redistribution with bilateral pleural fluid accumulation. Computed tomography (CT) imaging excluded aortic dissection, massive pulmonary embolism, pericardial fluid and retroperitoneal hematoma. After blood, urine and pleural fluid had been collected for culture, he was empirically treated intravenously with amoxicillin/clavulanate 2.2 g four times a day and ciprofloxacin 200 mg twice a day for presumed septic shock. A transthoracic echocardiography, that was performed because of the systolic murmur and the hemodynamic instability, showed evidence of tricuspid valvular involvement with several large vegetations of approximately 2.5 cm in diameter, severe valvular regurgitation, and a reduced ejection fraction (45%) (Figure 1). As no pathogen could be isolated from blood cultures after 60 hours of incubation, we considered all agents of culture-negative endocarditis as possible etiology. Investigations for HACEK microorganisms, and serologic studies for *Bartonella spp*., *Brucella spp*. and *Coxiella burnetti *were negative; PCR of the blood and pleural fluid for *Tropheryma whipplei *was positive. The PCR technique described by Meibach *et al*. in 2003 was used [11]. Having diagnosed *Tropheryma whipplei *right heart endocarditis, we switched the antibiotic regimen to ceftriaxone 2 g once daily. Favorable clinical changes kept him from requiring surgery, and he returned home after 25 days with a combined treatment of doxycycline 100 mg twice a day, hydroxychloroquine 200 mg and sulfamethoxazole-trimethoprim 160/800 mg three times a day for a minimum of one year. The blood levels of doxycycline and hydroxychloroquine were measured every other month and doses adapted to therapeutic levels (doxycycline: > 5 μg/ml, hydroxychloroquine 1 +/- 0.2 mg/l). At a one-year follow-up he had completely recovered, gained weight and all his laboratory values were back to normal. A control echography performed after one year (Figure 2) confirmed the treatment success.

## Discussion

Right-sided endocarditis, which usually involves the tricuspid valve, occurs predominantly in intravenous drug users or is related to congenital defects, intracardiac catheters, pacemakers or cardiac anomalies [12]. Physicians often use the Duke criteria to diagnose endocarditis, but in patients with blood culture-negative endocarditis due to *Tropheryma whipplei*, two of the criteria (fever and a history of valvulopathy) are generally absent, making them difficult to diagnose [4]. In 2001, Fenollar *et al*. reviewed the literature of Whipple's endocarditis based on valve histology [5]. According to that study, patients with Whipple's endocarditis have no previous heart disease and are most often afebrile, their blood cultures are negative, and vegetation is observed on an echocardiograph in 75% of cases. Fenollar *et al *described 35 cases which came from a pathology series without detailed clinical history. A tricuspid endocarditis associated with involvement of other valves (mostly aortic) is reported in 6% of cases [4]. To the best of our knowledge, only one case of a patient diagnosed with *Tropheryma whipplei *tricuspid endocarditis without any other valve involved has been completely reported [13]. It describes the case of a young female presenting with migratory arthralgia, abdominal pain, diarrhea, and weight loss of two years duration. Physical examination revealed a systolic murmur on the left sternal margin. The diagnosis of Whipple's disease was made on jejunal biopsy by electron microscopy and transoesophageal echocardiogram revealed a fixed vegetation on the tricuspid valve. The patient was successfully treated with penicillin G and streptomycin for 14 days, followed by sulfamethoxazole-trimethoprim for one year [13]. No surgery involving the valve was carried out.

Contrary to the patient described by Ferrari *et al*. who presented symptoms (arthralgia and digestive involvement) suggestive of Whipple's disease [13], our patient presented a *Tropheryma whipplei *endocarditis manifesting as severe shock. Apart from weight loss, he didn't exhibit any of the typical symptoms of Whipple's disease. He also did not have any risk factors for right-sided endocarditis.

Diagnosis of Whipple's disease is suspected most of the time on the basis of gastrointestinal symptoms and is generally confirmed by intestinal biopsies. According to recently published data it seems that the occurrence of endocarditis due to *Tropheryma whipplei*, without any of the classical features of Whipple's disease, is not as rare as was previously thought [14]. As we did not suspect Whipple's disease at the beginning, we did not perform intestinal biopsies. No serology is yet available. PCR is especially useful for the diagnosis of Whipple endocarditis and may be directly performed on blood samples and pleural fluid, as we did, or on valvular samples [15]. PCR performed on blood allows a non-invasive diagnosis and rapid results. However, cautious interpretation of PCR results is needed since PCRs have been positive in healthy patients, most likely as a result of contamination [16]. Conversely, sensitivity of PCR on blood samples may be impaired by the presence of PCR inhibitors and by the relatively low amount of circulating DNA. For patients with concomitant gastrointestinal involvement, diagnosis may also be made more easily from a small bowel biopsy that will be positive on PAS-staining. In the present case, the obvious vegetation on cardiac ultrasound, the positive PCR on two different samples (blood and pleural fluid), and the favorable change in the condition with antibiotics makes the etiological role of *Tropheryma whipplei *in this right-sided endocarditis absolutely clear.

Concerning treatment, our patient was initially treated by ceftriaxone then with a combination of sulfamethoxazole/trimethoprim, hydroxychloroquine and doxycycline for one year. By analogy with what is known about *Coxiella burnetii*, the association of a lysotropic agent (hydroxychloroquine) to doxycycline tends to reduce the acidity of the vacuole in which *Tropheryma whipplei *is located and thus improves the efficacy of doxycycline inactive at lower pH [17,18]. Interestingly, between sulfamethoxazole and trimethoprim, only sulfamethoxazole is active and trimethoprim is absolutely not effective against *Tropheryma whipplei*; thus, the association of sulfamethoxazole and trimethoprim represents a monotherapy.

## Conclusion

In summary, *Tropheryma whipplei *infectious endocarditis is a rare disease and tricuspid involvement is found even less often. This diagnosis should always be considered when facing a blood-culture negative endocarditis particularly in right-sided endocarditis without risk factors. Although not standardized yet, treatment of *Tropheryma whipplei *endocarditis should probably include a bactericidal antibiotic (such as doxycycline) and should be given for a prolonged period of time (a minimum of one year).

## Competing interests

The authors declare that they have no competing interests.

## Consent

Written informed consent was obtained from the patient for publication of this case report and any accompanying images. A copy of the written consent is available for review by the Editor-in-Chief of this journal.

## Authors' contributions

VG was responsible for writing the manuscript and reviewing the literature. ZG, SJ and RR had significant roles in data gathering and were major contributors to the content of the manuscript. VG, ZG, RR, GG and DG were involved in patient management. GG and DG had a significant role in data interpretation and provided significant revisions to the manuscript. All authors read and approved the final manuscript.
